# Cellular senescence: from pathogenic mechanisms to precision anti-aging interventions

**DOI:** 10.18632/aging.206375

**Published:** 2026-05-04

**Authors:** Jian Deng, Ruipu Sun, Zhiyong Bai, Lisha Fang, Xudong Zhao, Dong Yang

**Affiliations:** 1 Department of Targeting Therapy and Immunology and Laboratory of Cell Engineering and Immunotherapy, Cancer Center and State Key Laboratory of Respiratory Health and Multimorbidity and Frontiers Science Center for Disease-Related Molecular Network, West China Hospital, Sichuan University, Chengdu, Sichuan, China.

**Keywords:** cellular senescence, aging mechanisms, functional heterogeneity, precision anti-aging

## Abstract

For decades, research on cellular senescence has predominantly focused on the static identification of senescent cells using markers such as p16 and β-galactosidase, largely overlooking their functional heterogeneity across spatiotemporal dimensions. Accumulating evidence reveals that senescent cells are not merely deleterious pathological byproducts; rather, a subset plays indispensable physiological roles in embryonic development, wound healing, and the maintenance of tissue homeostasis. Based on these insights, this review summarizes the induction mechanisms of cellular senescence and the subsequent evolution of their functional phenotypes across diverse tissues. Consequently, we propose a novel paradigm for senescence management centered on “prevention first, followed by precision intervention.” This strategy involves, on one hand, mitigating environmental stressors and optimizing metabolism to intercept the onset of detrimental senescence at its source. On the other hand, it advocates for the functional profiling of existing senescent populations via single-cell omics and lineage tracing, enabling the targeted clearance of “maladaptive” components that drive pathological phenotypes while preserving “beneficial” elements essential for physiological stability. Such a systematic intervention, grounded in the classification of induction factors and functional subtypes, offers a safer and more efficacious trajectory for the prevention of age-related diseases and the extension of healthspan.

## INTRODUCTION

Cellular senescence, characterized by a state of stable cell-cycle arrest, has evolved from an initial observation in *in vitro* experiments to a central theoretical pillar for understanding systemic aging and age-related pathological processes. The traditional research paradigm primarily relies on a suite of consensus biomarkers for the identification of senescent cells. Among the most representative are the cyclin-dependent kinase inhibitors p16^INK4a^ and p21^CIP1^, the activity of senescence-associated beta-galactosidase (SA-β-gal), and the DNA damage [1–6]. By adopting a biomarker-based definitional model, we gain a critical instrument to map the landscape of senescent cell accumulation, offering preliminary insights into its mechanistic associations with dysfunction across multiple organ systems [7–9]. In fact, senescent cells are not a homogeneous group but exhibit profound functional heterogeneity. Senescent cells with similar molecular phenotypic characteristics, regulated by their cell origin, tissue microenvironment, and induction background, may have vastly different or even opposite effects on tissue homeostasis. For example, senescent glial cells in the brain have been proven to be key factors driving neuroinflammation and cognitive decline [10, 11], but senescent pancreatic β cells display superior insulin secretory capacity relative to younger cells, representing a distinct functional shift during the aging process [12]. This insight has catalyzed a pivotal paradigm shift: moving beyond rudimentary “senescence profiling” to a mechanistic dissection of the functional trajectories of discrete senescent subpopulations. Emerging evidence increasingly highlights that certain senescent cohorts are not merely deleterious bystanders but active rheostats of tissue homeostasis, characterized by profound context-specificity and functional pleiotropy [13, 14].

Elucidating the inductive mechanisms of cellular senescence constitutes the cornerstone for implementing effective clinical interventions, centered on a “prevention-over-cure” paradigm. The primary objective is upstream intervention: maintaining genomic stability, mitigating oxidative damage, and modulating canonical pro-senescent signaling axes (e.g., p53/p16) to delay the onset of the senescence program and restrict the systemic accrual of senescent cells [8]. Concurrently, the deepening characterization of senescent heterogeneity is driving a paradigm shift from “generalized ablation” toward “precision targeting.” Given that distinct senescent subpopulations exert transient physiological benefits during wound healing and tissue repair [15]. Early anti-aging strategies that targeted senescent cells based on senescence “state markers” (e.g., p16, SA-β-gal) rather than “functional markers” significantly lacked precision and carried the potential risk of disrupting tissue homeostasis. [13, 14, 16]. Consequently, the cutting edge of the field has shifted toward precision senolytics aimed at the selective ablation of deleterious senescent cell subsets. The challenge lies in preserving 'beneficial' senescent cells and healthy tissues, which requires the discovery of specific surface markers or signaling conduits that differentiate cellular outcomes. Such advancements will establish the necessary framework for highly targeted and individualized anti-aging strategies. The therapeutic landscape has evolved from first-generation small molecules targeting pro-survival pathways (e.g., Dasatinib, Quercetin, and Fisetin) [17–20], to highly sophisticated second-generation modalities. These include immunological interventions, such as chimeric antigen receptor (CAR) T cells engineered to recognize senescence-associated surface antigens (e.g., Urokinase-type plasminogen activator receptor (uPAR) or natural killer group 2D ligands (NKG2DL)) [21–23], and senomorphics, which neutralize or suppress the senescence-associated secretory phenotype (SASP) without necessitating cell death [24, 25]. In conclusion, the distinct profiles of pharmacodynamics, target specificity, and off-target risks across intervention modalities underscore the imperative for matching therapeutic mechanisms with specific senescent landscapes and histopathological features during clinical translation.

The field of precision geroprotection currently confronts several critical bottlenecks, primarily concerning the characterization of robust biomarkers, the engineering of targeted delivery modalities, and the mechanistic elucidation of the aging trajectory. A major impediment is the deficiency in senescence-specific markers, which precludes the selective ablation of senescent cells; meanwhile, systemic administration frequently triggers off-target sequelae and adverse effects. Future research must prioritize the discovery of high-specificity markers, the development of stimuli-responsive delivery platforms, and the mapping of spatiotemporal senescence dynamics to establish a rigorous theoretical framework and optimal therapeutic windows for clinical translation [26, 27].

This review aims to systematically integrate the current landscape of research in the field of anti-aging. It begins by providing an in-depth analysis of the mechanisms underlying the formation of senescent cells across diverse tissues, tracing the conceptual evolution from conventional biomarker detection toward function-oriented characterization (Figure 1). Building upon this foundation, the article critically evaluates the burgeoning array of senolytic and senomorphic strategies, with a particular focus on their molecular modes of action, validated outcomes within major organ systems, and inherent technical constraints. Finally, the discussion centers on the core bottlenecks currently hindering translational medicine, offering a prospective outlook on the developmental trajectories required to achieve safe and efficacious clinical applications of future anti-aging therapies.

**Figure 1:**
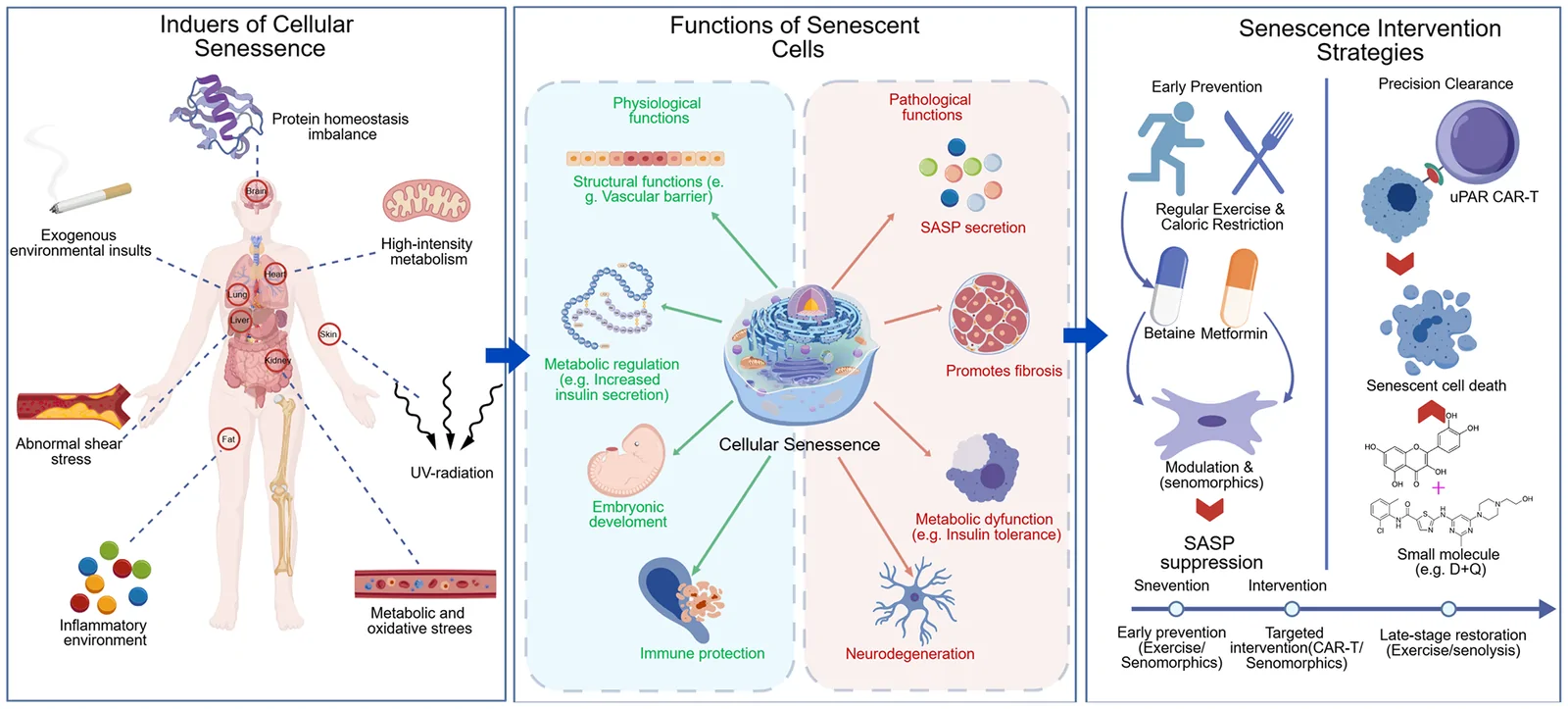
**From senescence induction to functional characterization and anti-aging interventions.** Tissue-specific senescence inducers (Left), dual roles of senescent cells (Middle), and anti-aging interventions (Right).

## Major inducing factors of cellular senescence in tissues

### Factors influencing the senescence of major liver cell types

Acting as a metabolic center, the liver represents one of the most thoroughly investigated organs in aging research. Under physiological aging, senescent cell counts in the liver increase incrementally with chronological age [13]. Research by Grosse et al. revealed marked cellular heterogeneity among p16-positive senescent cells in 12-month-old murine livers, with liver sinusoidal endothelial cells and CD45-positive immune cells (mainly F4/80-positive macrophages) accounting for roughly 70% and 30%, respectively [14]. This cellular landscape is mirrored under pathological stress. In CCl_4_-induced fibrosis and choline deficient amino acid-induced non-alcoholic steatohepatitis (NASH) models, the senescent cells are not restricted to endothelial cells (ECs) and macrophages but extends to a broad spectrum of cell types, including fibroblasts, hepatic stellate cells, mesenchymal cells, and various immune subsets such as T cells, B cells, and dendritic cells [13].

### Endogenous attrition and environmental stress: synergistic triggers of LSEC senescence

Liver sinusoidal endothelial cells (LSECs) are specialized endothelial cells that constitute the sinusoidal wall, serving as a pivotal barrier for material exchange between the blood and hepatocytes. The structural and functional integrity of LSECs is paramount for maintaining normal nutrient transport and oxygen exchange within the liver. Emerging evidence indicates that LSECs are among the cell populations most vulnerable to the natural aging process. The molecular mechanisms underlying LSEC senescence primarily stem from the synergistic effects of chronic endogenous depletion and exogenous stress. First, replicative senescence serves as the fundamental mechanism of LSEC degeneration. Throughout successive cell divisions, telomeres—the protective structures at chromosomal termini—undergo programmed shortening. Once a critical threshold is reached, a persistent DNA damage response is triggered, subsequently activating the canonical p53/p21 and p16/Rb signaling pathways to induce irreversible cell cycle arrest [28, 29]. Furthermore, epigenetic remodeling plays a crucial role. In aged or injured livers, the DNA methylation profiles and histone modification patterns of LSECs undergo significant shifts. These alterations consolidate the senescent phenotype by modulating the expression of genes associated with angiogenesis, inflammation, and fibrogenesis [30–32]. Second, metabolic and oxidative stress exacerbate the senescent progression of LSECs. Mitochondrial dysfunction leads to the excessive production of reactive oxygen species (ROS). When ROS concentrations surpass the cellular antioxidant defense threshold, widespread damage occurs to proteins, lipids, and genomic DNA. Given their direct exposure to portal venous blood, LSECs are chronically bombarded by pro-oxidative factors—including gut-derived lipids, alcohol metabolites, and endotoxins (e.g., lipopolysaccharide, LPS)—rendering them highly susceptible to severe oxidative stress [32–35]. Finally, alterations in hemodynamic properties represent a critical physical driver. In pathological contexts such as cirrhosis, elevated portal pressure results in aberrant shear stress within the hepatic sinusoids. This mechanical stress is transduced into intracellular biochemical signals via mechanosensors, such as integrins and ion channels, leading to dysregulated gene expression. LSECs subjected to chronic abnormal shear stress are more prone to exhibiting pro-inflammatory and pro-fibrotic characteristics [36–38].

Aging-driven morphological remodeling of LSECs is characterized by profound “de-fenestration” and “capillarization” [14, 39]. In the senescent state, the number of non-diaphragmatic fenestrae decreases, and their diameters shrink, accompanied by the aberrant deposition of the basement membrane. The emergence of a continuous collagen type IV-positive layer on the cell surface transforms the originally highly permeable hepatic sinusoids into continuous capillaries resembling those in systemic circulation. This morphological transition severely impairs hepatic filtration and material exchange efficiency. Consequently, it leads to the retention of triglyceride-rich lipoproteins in the plasma, exacerbating hyperlipidemia and hepatocellular lipid accumulation. Furthermore, it impedes the effective transport of insulin into the space of Disse, serving as a critical factor in inducing hepatogenic insulin resistance [34, 40, 41].

Secondly, senescent LSECs actively reshape the hepatic microenvironment by secreting the SASP [42]. Under physiological conditions, healthy LSECs inhibit the activation of hepatic stellate cells (HSCs) by synthesizing vasorelaxants such as nitric oxide (NO) and maintaining TGF-β1 in an inactive state [43]. In contrast, senescent LSECs transition toward a pro-fibrotic phenotype: their capacity for NO synthesis is compromised, while the secretion of vasoconstrictors (e.g., endothelin-1) increases. Concurrently, these cells release large quantities of activated TGF-β1 and overexpress plasminogen activator inhibitor-1 (PAI-1). As a hallmark SASP component, PAI-1 further exacerbates fibrogenesis by inhibiting the degradation of the extracellular matrix [21, 38], Additionally, inflammatory cytokines such as IL-6 and IL-1β secreted by senescent LSECs continuously recruit monocyte-derived macrophages and neutrophils, establishing a “senescence-inflammation” positive feedback loop [14, 39, 44, 45]. In the field of cancer biology, this senescent microenvironment has also been demonstrated to exert immunosuppressive effects, thereby promoting the initiation and progression of hepatocellular carcinoma [46].

Thirdly, senescence diminishes the supportive role of LSECs in liver regeneration. As core organizers of the regenerative microenvironment, healthy LSECs support hepatocyte proliferation by secreting angiocrine and growth factors, including vascular endothelial growth factor (VEGF), Wnt family member 2(Wnt2) and hepatocyte growth factor. The age-related downregulation of these regenerative factors significantly weakens the liver's repair capacity following partial hepatectomy or injury [14]. This represents one of the intrinsic mechanisms by which elderly populations and patients with chronic liver disease are more susceptible to progressing from liver injury to acute liver failure [47].

### From immunosurveillance to inflammaging: phenotypic evolution of KCs under multiple stressors

Kupffer cells (KCs), the resident macrophages of the liver, constitute a critical subset of the hepatic senescent cell population. Due to their direct exposure to portal venous blood, KCs are frequently challenged by gut-derived endotoxins, alcohol metabolites, and free fatty acids. These stimuli induce the production of high levels of ROS in KCs, which directly damage cellular macromolecules and activate signaling pathways such as NF-κB and p38 MAPK, thereby driving KCs into a state of inflammaging [48, 49]. In the pathological progression of NAFLD/NASH, KCs engulf vast quantities of apoptotic hepatocyte debris and oxidatively modified lipid droplets via the “lipid spillover” effect. The excessive intracellular accumulation of lipids, such as cholesterol and saturated fatty acids, triggers endoplasmic reticulum (ER) stress and mitochondrial dysfunction. These processes, coupled with the activation of the NOD-like receptor protein 3(NLRP3) inflammasome, synergistically induce cellular senescence [50]. Senescent KCs develop a robust SASP, which further deteriorates the hepatic metabolic milieu [51]. Furthermore, a positive feedback loop established between the chronic inflammatory environment and SASP continuously exacerbates the degree of senescence in KCs [52–55]. Persistent translocation of LPS into the liver, resulting from intestinal barrier dysfunction, also serves as a pivotal immunological driver of KC senescence via the toll-like receptor (TLR) 4-mediated chronic low-grade stress pathway [56]. Persistent translocation of LPS into the liver, resulting from intestinal barrier dysfunction, also serves as a pivotal immunological driver of KC senescence via the TLR4-mediated chronic low-grade stress pathway [13, 14]. The profound shifts in KC function impair the liver’s immune defense and regenerative potential, serving as a key driver propelling the progression from NAFLD/NASH to liver fibrosis, cirrhosis, and hepatocellular carcinoma.

### From fibrotic brake to pro-inflammatory source: revisiting the pathophysiological significance of HSC senescence

The senescence of HSCs presents a unique “paradoxical” character in liver pathophysiology. While acting as a physiological brake to limit excessive fibrogenesis, the resulting SASP creates a microenvironment that paradoxically promotes chronic liver disease and hepatocarcinogenesis. Distinct from LSECs and KCs, the hallmark of HSC senescence is the phenotypic transition from activated, proliferating, and matrix-secreting myofibroblasts to a state of cell cycle arrest coupled with hypersecretory activity [52]. During the initial stages of liver injury, quiescent HSCs are activated and transition into proliferative myofibroblasts, which secrete extracellular matrix (ECM) to shield hepatic tissue from further insult. However, this sustained and intensive proliferation accelerates telomere attrition, eventually triggering a replicative senescence program. This senescence-induced permanent cell cycle arrest substantially curtails the production of ECM components like collagen, thereby limiting myofibroblast expansion and the progression of fibrosis at its source. [52, 57]. Furthermore, senescent HSCs highly express “find-me/eat-me” signals, such as UL16 binding protein 2, which facilitate their specific recognition and clearance by natural killer cells. Nevertheless, if the generation of senescent HSCs exceeds the capacity of the immune system to clear them, the persistent accumulation of these cells fuels a pro-fibrotic microenvironment through SASP, ultimately intensifying inflammatory responses and tissue scarring. Exposure of HSCs to oxidative stress and genotoxic insults can trigger DNA double-strand breaks, which subsequently induce stress-induced premature senescence via the activation of the ATM/ATR-Chk1/2-p53/p21 signaling axis. This form of cellular damage is characteristically observed in response to ROS within the hepatic inflammatory milieu, stimulation by chemotherapeutic agents, and the detrimental impact of alcohol metabolites [58]. Concurrently, senescent HSCs develop a robust SASP. The resulting secretome comprising proinflammatory cytokines (e.g., IL-6, IL-1β), chemokines (e.g., CCL2, CXCL1), and tissue remodeling factors (e.g., VEGF, MMPs) exerts multifaceted effects: it recruits diverse immune cell populations to exacerbate chronic inflammation while simultaneously functioning as a pivotal driver for the initiation and progression of hepatocellular carcinoma [59, 60].

### Hepatocyte senescence and functional alterations.

As the primary functional units responsible for metabolism, biosynthesis, and detoxification, hepatocytes undergo senescence as a pivotal driver of hepatic functional decline. Under pathological conditions such as metabolic dysfunction-associated steatohepatitis (MASH), the accumulation of free fatty acids, cholesterol, and lipotoxic metabolites (e.g., ceramides) collectively triggers hepatocyte senescence by inducing oxidative stress, ER stress, and mitochondrial dysfunction [61, 62]. At the functional level, senescent hepatocytes exhibit a diminished capacity for synthesizing albumin and coagulation factors, reduced activity of drug-metabolizing enzymes (notably the CYP450 family), and impaired fatty acid oxidation and triglyceride export, which further exacerbates intrahepatic lipid deposition [63]. From a regulatory perspective, senescent hepatocytes secrete the IL-6, IL-8, PAI-1, and TGF-β, which drives the activation of HSCs and the capillarization of LSECs. Furthermore, the recruitment of immune cells by these SASP components serves as a critical catalyst in the progression from NAFLD to NASH and subsequent fibrosis. Indeed, evidence suggests that the targeted clearance of senescent cells can effectively reverse age-related hepatic steatosis [28, 64]. Beyond hepatocytes, the senescence of cholangiocytes [65], and non-macrophage immune cells [13, 66] also contributes synergistically to the global aging process of the liver.

### Factors influencing the senescence of major lung cell types

Lung senescence represents a fundamental mechanistic driver underlying respiratory decline and the pathogenesis of diverse chronic pulmonary pathologies, including idiopathic pulmonary fibrosis (IPF), chronic obstructive pulmonary disease (COPD), and lung adenocarcinoma. Owing to the lung's relentless exposure to exogenous environmental insults—such as cigarette smoke, particulate matter, and diverse pathogens, the persistent induction of oxidative stress and genomic instability (DNA damage) constitutes the primary etiology of cellular senescence within the pulmonary niche.

### Alveolar epithelial senescence: environmental triggers and pathological remodeling

The alveolar epithelium serves as a critical physiological barrier for gas exchange, comprising alveolar type I (AT1) cells, specialized for gas diffusion, and alveolar type II (AT2) cells, which secrete pulmonary surfactants and function as the resident progenitor pool for AT1 cells. During pulmonary ventilation, direct exposure to exogenous stressors, including cigarette smoke, particulate matter (PM2.5), and ozone—triggers robust DNA damage, acting as primary pathological catalysts for COPD and pulmonary fibrosis [67–69]. Emerging evidence underscores that AT2 cells in patients with IPF frequently exhibit profound telomere attrition, which subsequently triggers a terminal senescence program; importantly, these senescent AT2 cells undergo a deleterious loss of their proliferative capacity and multilineage differentiation potential, thereby compromising the effective restoration of damaged alveolar architecture. This regenerative failure culminates in either alveolar simplification (emphysema) or aberrant fibroproliferative repair [70]. IL-6, IL-1β, TGF-β, and PAI-1 secreted by other senescent cells influence the trajectory of disease progression, ultimately leading to tissue destruction or refractory fibrosis [67, 70]. Notably, TGF-β operates as a master profibrotic rheostat, driving the activation of resident fibroblasts and the excessive deposition of the ECM [71].

### Senescence and functional dichotomy of pulmonary fibroblasts

Pulmonary fibroblasts are quintessential orchestrators of interstitial homeostasis. Upon lung injury, these cells are conventionally activated into myofibroblasts to facilitate tissue repair. Similar to hepatic stellate cells, the senescence program in lung fibroblasts exerts a complex, dual-edged effect on tissue remodeling. In the context of persistent injury, aberrant proliferative surges can lead to telomere attrition, culminating in replicative senescence. Conversely, paracrine signaling from neighboring senescent epithelial cells, predominantly mediated by SASP components such as TGF-β can bypass the requirement for extensive replication to trigger stress-induced premature senescence in fibroblasts [69]. From a homeostatic feedback perspective, the transition of myofibroblasts into a senescent state serves as an intrinsic brake on fibrogenesis. By entering permanent cell-cycle arrest, these senescent cells exhibit a diminished capacity for collagen synthesis alongside an upregulation of matrix-degrading enzymes, thereby constituting a critical negative feedback loop that limits excessive scarring. Paradoxically, the fibroblast-derived SASP is enriched with MMPs and a distinct repertoire of pro-inflammatory cytokines. While these secretomes facilitate the clearance of aberrant extracellular matrix, they simultaneously jeopardize the structural integrity of the normal alveolar scaffold and sustain a state of chronic low-grade inflammation. This persistent inflammatory milieu effectively remodels the lung microenvironment into a pro-tumorigenic niche, fostering the development of secondary malignancies [70, 72].

### Exogenous and endogenous triggers of lung endothelial senescence: from environmental stress to oxidative damage

The expansive and dense pulmonary capillary network renders endothelial cells (ECs) exceptionally vulnerable to exogenous and endogenous insults, including cigarette smoke [73–75], chronic hypoxia [76, 77], and systemic inflammatory cytokines [78, 79]. These stressors collectively trigger robust oxidative stress and genomic instability, ultimately driving ECs into a state of senescence [79, 80]. Senescent ECs compromise the integrity of the alveolar-capillary barrier, leading to heightened vascular permeability, inflammatory exudation, and subsequent tissue edema [70, 81], Concurrently, a decline in NO bioavailability coupled with the aberrant elevation of endothelin-1 orchestrates a shift toward a pro-constrictive phenotype, facilitating the development of pulmonary hypertension. Furthermore, senescent ECs actively recruit leukocytes through the high expression of cell adhesion molecules, such as vascular cell adhesion molecule 1(VCAM-1) and intercellular adhesion molecule 1(ICAM-1) [82, 83], A hallmark feature of the endothelial senescent secretome is the profuse secretion of PAI-1, the expression of which is stringently regulated by the p53-p21 and NF-κB signaling axes [84, 85]. Elevated levels of PAI-1 antagonize tissue-type plasminogen activator, thereby impairing plasmin generation and fibrinolytic efficiency [86–88]. This pro-coagulant shift not only exacerbates intra-pulmonary inflammation but also significantly heightens the risk of pulmonary microthrombosis, contributing to the systemic and local failure of lung perfusion.

### Environmental stress and oxidative insult: the catalysts for alveolar macrophage dysfunction

Alveolar macrophages (AMs), the predominant resident immune sentinels of the respiratory tract, serve a pivotal role in maintaining immunological surveillance and homeostatic clearance. Their transition into senescence is driven by a complex interplay between cell-intrinsic genetic factors and extrinsic environmental insults. With advancing age or chronic pathological stimulation, the epigenetic landscape of AMs, characterized by DNA methylation patterns and histone modifications undergoes profound remodeling. This epigenetic shift leads to a dysregulated pro-inflammatory transcriptional program, constraining macrophages into a persistent, low-grade inflammatory phenotype. This phenomenon, often termed “inflammaging,” is considered a primary driver of chronic pulmonary inflammation [89]. In parallel with other pulmonary cell types, AMs are continuously exposed to inhaled noxious agents, including cigarette smoke, airborne pollutants (e.g., PM2.5), and mineral dust. These particulates trigger a burst of ROS, inflicting cumulative oxidative damage to lipids, proteins, and genomic DNA [90]. Such oxidative stress, coupled with persistent inflammatory insults, induces mitochondrial DNA mutations and a loss of membrane potential, establishing a self-perpetuating cycle of mitochondrial dysfunction [91]. Furthermore, the proteostatic machinery, comprising the ubiquitin-proteasome and autophagy-lysosome systems, becomes compromised in senescent AMs. The subsequent accumulation of misfolded proteins and damaged organelles disrupts cellular metabolic equilibrium, culminating in functional exhaustion [92]. Critically, the chronic activation of the NLRP3 inflammasome exacerbates the release of IL-1β and IL-18, reinforcing the pro-inflammatory microenvironment and accelerating the kinetics of macrophage senescence [93–96]. These molecular alterations are synchronized with the upregulation of cyclin-dependent kinase inhibitors, such as p16INK4a and p21CIP1, which ultimately lock the cells into an irreversible state of senescence [97].

### Factors driving cellular senescence in major renal cell populations

### High metabolic demand and vulnerability: mechanisms of tubular epithelial cell senescence

Renal tubular epithelial cells (TECs) are tasked with immense reabsorptive and secretory workloads, rendering them exquisitely sensitive to cellular injury due to their high metabolic demand. Oxidative stress, telomere attrition, and genomic instability are the primary drivers of TEC senescence [98]. Telomere exhaustion, alongside ROS-induced non-telomeric DNA double-strand breaks, is prevalent within the tubular compartment. These lesions orchestrate a persistent cell-cycle arrest primarily through the activation of the ATM–Chk2–p53 signaling axis [98]. Concomitantly, the basal autophagic flux in renal tubules declines significantly with advancing age, leading to the impaired clearance of damaged organelles and proteotoxic aggregates. Targeted deletion of essential autophagy genes, such as Atg5 or Atg7, specifically within the tubular compartment has been shown to precipitously accelerate cellular senescence, triggering robust interstitial fibrosis and profound loss of renal function [99]. Senescent TECs are further characterized by aberrant mitochondrial morphology and augmented ROS generation [100]. This mitochondrial dysfunction is compounded by defects in PINK1/Parkin-mediated mitophagy, resulting in the intracellular accumulation of dysfunctional mitochondria that amplify oxidative collateral damage [101]. This cascade of events has been identified as a cornerstone of tubular injury in both age-related nephropathy and the early stages of diabetic kidney disease.

### Metabolic stress and ECM remodeling: pathogenic aging of glomerular cells

Podocytes. As terminally differentiated cells, podocytes possess extremely limited regenerative potential. Metabolic insults (such as hyperglycemia) and mechanical stress (such as glomerular hypertension) trigger the aberrant activation of the mTORC1 signaling pathway, which serves as a critical catalyst for podocyte senescence. In senescent podocytes, the downregulation of mitochondrial fusion proteins, notably Mfn2, further exacerbates energetic insufficiency [102]. This is followed by the diminished expression and spatial disorganization of slit diaphragm-associated proteins, including nephrin and podocin, which directly compromises the integrity of the glomerular filtration barrier. Such structural deterioration manifests as proteinuria and precipitates the progression toward glomerulosclerosis [103]. Mesangial Cells. The drivers of senescence in mesangial cells are distinct and context-dependent. Under diabetic conditions, hyperglycemia and the accumulation of advanced glycation end-products (AGEs) drive mesangial cells into replicative senescence by inducing ROS-mediated DNA damage. Senescent mesangial cells undergo a profound phenotypic shift, characterized by the excessive deposition of ECM components, such as type IV collagen and fibronectin. Concurrently, these cells adopt a proinflammatory secretome that promotes glomerular basement membrane thickening and mesangial expansion, ultimately culminating in irreversible glomerular sclerosis [104, 105].

### Senescence and functional remodeling of renal interstitial fibroblasts

The senescence of interstitial fibroblasts is orchestrated by a paracrine signaling axis initiated by injured or senescent renal tubular epithelial cells (RTECs). These dysfunctional RTECs release profibrotic secretomes, including TGF-β and connective tissue growth factor, which trigger a senescent transition in neighboring interstitial fibroblasts [105]. The functional repercussions of fibroblast senescence appear to be dichotomous. On one hand, the marked reduction in proliferative capacity inherent to the senescent state may serve as a localized brake, potentially constraining the excessive fibroproliferative expansion typically observed in progressive fibrosis. On the other hand, senescent fibroblasts exert profound deleterious effects on the interstitial microenvironment. By secreting a complex repertoire of MMPs and tissue inhibitors of metalloproteinases, these cells disrupt the homeostatic balance between the biosynthesis and degradation of the ECM. Furthermore, via the secretion of proinflammatory cytokines such as IL-6 and monocyte chemoattractant protein-1, senescent fibroblasts actively recruit and prime immune cells, thereby sustaining a chronic inflammatory milieu within the renal interstitium [106].

### Metabolic stress and exogenous stimuli: triggers of macrophage senescence

Resident renal macrophages are chronically exposed to a hostile milieu characterized by persistent inflammation and metabolic waste accumulation. In the context of diabetic nephropathy, for instance, AGEs ligate their receptor RAGE, subsequently activating the NF-κB signaling pathway and the NLRP3 inflammasome to trigger cellular senescence [107, 108]. Hyperglycemia, acting as a systemic stressor, can directly induce a senescent transition in bone marrow-derived macrophages (BMDMs). Strikingly, the adoptive transfer of these senescent macrophages into recipient mice exacerbates renal injury, underscoring the mechanistic link between macrophage senescence and diabetic disease progression [109]. Furthermore, exogenous nephrotoxic insults not only damage renal tubular epithelial cells directly but also serve as potent triggers for macrophage senescence. This “multicellular senescence” synchrony significantly amplifies the severity of renal parenchymal damage [44, 110, 111]. The functional repertoire of senescent macrophages is profoundly impaired. These cells lose their essential “homeostatic scavenger” capabilities, failing to effectively efferocytosis apoptotic cells or clear immune complexes and metabolic debris. The consequent accumulation of these undegraded substrates leads to secondary tissue necrosis and triggers a self-perpetuating immune response [112, 113]. Central to the pathogenic potential of senescent macrophages is the sustained release of a SASP. By secreting a potent cocktail of proinflammatory and profibrotic cytokines, alongside the dysregulated expression of MMPs, senescent macrophages fundamentally destabilize the renal microenvironmental rheostat [96].

### Factors influencing the senescence of major cardiac cell types

As a metabolically demanding and structurally sophisticated organ, the heart’s systemic dysfunction stems from the programmed degeneration of cardiomyocytes, fibroblasts, and endothelial cells. This decline is orchestrated by the deleterious interplay of advancing age, metabolic perturbations, and chronic low-grade inflammation, a phenomenon termed “inflammaging” [114].

### Mitochondrial dysfunction and DNA damage: core drivers of cardiomyocytes cellular aging

Cardiomyocytes (CMs), the fundamental contractile units of the heart, exhibit senescence signatures primarily characterized by the loss of proteostatic and metabolic homeostasis alongside hypertrophic remodeling. Given their exceptional mitochondrial density, the accumulation of mitochondrial DNA (mtDNA) mutations and the progressive erosion of oxidative phosphorylation efficiency serve as core drivers of CM senescence [115]. Although CMs possess negligible proliferative capacity, critical telomere attrition and persistent DNA damage response can still converge on the p53/p16INK4a signaling axes, forcing these post-mitotic cells into a senescent-like state [116]. Furthermore, the heart’s role as the central hemodynamic pump renders it vulnerable to mechanical stress. Chronic pressure overload, stemming from systemic hypertension or aortic stenosis, induces cell-cycle arrest and triggers a robust SASP via sustained mechanical stretch. Ultimately, CM senescence manifests as maladaptive hypertrophy, impaired contractility, and bioenergetic failure, collectively establishing the cellular cornerstone for the pathogenesis of heart failure.

### Paracrine signaling and the “senescence spreading” effect in the cardiac microenvironment

Following myocardial infarction or hypertensive injury, cardiac fibroblasts (CFs) undergo persistent activation fueled by TGF-β, IL-11, and persistent mechanical strain. This process, coupled with replicative stress and oxidative damage, eventually precipitates replicative senescence [70, 117]. Beyond intrinsic factors, CF senescence is orchestrated by the local microenvironment; SASPs, including IL-1α and ROS, secreted by adjacent senescent cardiomyocytes and endothelial cells act via paracrine signaling to propagate a “senescence-spreading” effect throughout the myocardial interstitium [118]. Once transitioned into a secretory senescent state, these fibroblasts contribute to a deleterious feedback loop by continuously releasing profibrotic SASP components, such as excessively cross-linked collagens and MMPs. This shift from a transient reparative role to a chronic senescent phenotype directly drives interstitial fibrosis and myocardial stiffening, fundamentally contributing to the maladaptive remodeling of the failing heart.

### Mechanical and metabolic drivers of endothelial cell senescence

EC senescence is predominantly orchestrated by the synergistic interplay between systemic hemodynamic forces and circulating metabolic insults. From a mechanobiological perspective, disturbed flow patterns, characterized by non-laminar shear stress at arterial bifurcations, serve as a critical trigger for the induction of the endothelial pro-senescence program [119]. This mechanical vulnerability is further exacerbated by metabolic risk factors; chronic hyperglycemia and oxidized low-density lipoprotein (ox-LDL) directly inflict macromolecular damage, thereby initiating cellular senescence [82]. Senescent ECs transition into a distinct pro-inflammatory and pro-thrombotic state, characterized by a significant impairment in vasomodulatory capacity most notably a reduction in NO bioavailability [120]. This loss of homeostatic signaling not only compromises vascular elasticity but also acts as a primary mediator of age-related vascular remodeling and systemic arterial stiffening.

### Senescence in adipose tissue: a multicellular pathological network

As a central hub for energy homeostasis and endocrine regulation, adipose tissue undergoes a sophisticated aging process. This senescence is not a simple, cell-autonomous decline but rather a systemic pathological network orchestrated by the functional dysregulation of mature adipocytes, the aberrant polarization of resident macrophages, and the impaired regenerative potential of adipose progenitor cells.

### Adipocyte senescence and functional dysmetabolism

Chronic caloric excess drives pathological adipocyte hypertrophy, which subsequently triggers localized microenvironmental hypoxia, ER stress, and mitochondrial dysfunction. The resultant surge in ROS activates the DNA damage response and the p53/p16 signaling cascades, thereby consolidating and maintaining the senescent state. Furthermore, the overflow of free fatty acids and the ectopic deposition of triglycerides exert potent lipotoxic damage [121]. From an endocrine perspective, both systemic and locally-derived pro-inflammatory cytokines, most notably TNF-α and IL-6, directly perturb the insulin receptor substrate signaling pathway, fostering a state of “metabolic senescence.” Concurrently, age-associated fluctuations in systemic glucocorticoids and catecholamine levels significantly attenuate the regulatory efficiency of lipolysis and metabolic flexibility [122].

### The DAMPs-TLRs axis: initiation of senescence

Adipose tissue macrophages (ATMs) serve as the master rheostats of the local inflammatory microenvironment. Their senescent signature is characterized by the acquisition of a persistent pro-inflammatory phenotype and a concomitant decline in phagocytic clearance. Lipidic components and cellular debris released from senescent or necrotic adipocytes act as damage-associated molecular patterns, which chronically engage Toll-like receptors (TLRs) and other pattern recognition receptors. This persistent activation eventually precipitates macrophage exhaustion, driving them into a senescence-like state. Under physiological conditions, the adipose niche is predominantly populated by anti-inflammatory M2-like macrophages. However, the aging microenvironment, defined by chronic hypoxia and elevated pro-inflammatory tonus, orchestrates a polarizing shift toward a pro-inflammatory M1-like state, thereby establishing a critical “metabolic inflammatory amplifier” [18, 123]. Furthermore, SASP factors derived from neighboring senescent cells, such as CCL2 and IL-6, exert potent chemotactic effects that recruit circulating monocytes. These recruited cells undergo “pro-inflammatory education” within the dysfunctional niche, creating a feed-forward loop that accelerates the intraparenchymal accumulation of senescent cells.

### Replicative senescence and differentiation dysfunction in adipose progenitors

Adipocyte progenitor cells (APCs), or adipose-derived stem cells (ASCs) residing within the stromal vascular fraction, are the primary orchestrators of adipose tissue regeneration and remodeling. To meet the heightened demand for cellular turnover induced by aging or chronic overnutrition, these progenitors undergo accelerated proliferation. This sustained replicative stress triggers telomere attrition and culminates in replicative senescence. Senescent APCs exhibit a profoundly impaired adipogenic potential, undermining the tissue's capacity for healthy “hyperplastic expansion.” Instead of differentiating into functional, insulin-sensitive adipocytes, these dysfunctional progenitors undergo a “fibrogenic shift,” actively promoting interstitial fibrosis. This differentiation block inevitably leads to the spillover of excess lipids and their ectopic deposition in non-adipose organs such as the liver and skeletal muscle—a process that exacerbates systemic metabolic derangement and insulin resistance [122, 124].

To conclude, senescence within adipose tissue is characterized by a vicious cycle comprising three pivotal pillars: cellular hypertrophy, low-grade meta-inflammation, and impaired regenerative capacity. Nutritional surplus acts as the primary inciting factor for this process. Crucially, the paracrine dialogue among senescent populations, orchestrated by the SASP cascade, represents the fundamental molecular mechanism that propagates and maintains this pathological milieu, ultimately driving the progression of metabolic aging.

### Factors influencing aging in major brain cells

Aging in the brain is a complex process involving both intrinsic neuronal deterioration and the loss of homeostasis in the glial microenvironment. These intercellular interactions are critical drivers of neurodegenerative disease progression.

### Mitochondrial decay and bioenergetic insufficiency

Due to high metabolic requirements, neurons are highly susceptible to energy deficits. Age-related mitochondrial DNA mutations impair ATP production and trigger oxidative stress through ROS accumulation. The failure of the autophagy-lysosome and ubiquitin-proteasome pathways results in the buildup of misfolded proteins, such as Aβ and tau. These aggregates disrupt synaptic plasticity and induce neurodegeneration [125, 126]. Additionally, altered DNA methylation and histone modifications suppress the expression of plasticity-related genes [8, 127, 128]. Decreased levels of survival factors, such as brain-derived neurotrophic factor, combined with dysfunctional synaptic inputs from adjacent senescent neurons, further accelerate the degenerative process [129].

### Metabolic overload and mitochondrial exhaustion

Astrocytes are essential for maintaining ionic homeostasis, neurotransmitter balance, and blood-brain barrier integrity. The metabolic support provided to neurons, particularly in glutamate processing, subjects astrocytes to high oxidative stress, resulting in mitochondrial damage. Pathological proteins like Aβ and tau activate inflammatory pathways, driving astrocytes into a senescence-associated reactive state [130, 131]. Aging-induced calcium signaling abnormalities disrupt the communication between astrocytes, neurons, and vessels, leading to impaired neurovascular coupling and inadequate cerebral blood flow regulation [132].

### Microglial senescence and neuroinflammation

Microglia are the primary immune cells of the brain. Their senescence drives neuroinflammation, characterized by impaired surveillance and reduced phagocytosis despite an exaggerated inflammatory response. Persistent exposure to abnormal proteins and DAMPs triggers epigenetic and metabolic reprogramming, shifting microglia into a dysfunctional state similar to aged peripheral macrophages. Decreased phagocytic activity leads to the accumulation of cellular debris and Aβ plaques [133, 134]. Additionally, SASP factors from senescent neurons and astrocytes maintain microglia in a pro-inflammatory state [135]. Recent studies highlight the critical role of senescent cells in neurodegenerative diseases, though the exact mechanisms linking senescence to disease onset remain a key focus of current research.

### Factors influencing senescence in major skin cells

The skin serves as the body’s first physical barrier. Its aging process is regulated by extrinsic factors, such as UV radiation and environmental pollution, alongside intrinsic genetic mechanisms. These factors lead to a systematic decline in the functional integrity of both the dermis and epidermis.

### UV-Induced DNA damage and cell cycle arrest

Ultraviolet radiation induces DNA damage, including the formation of cyclobutane pyrimidine dimers, and triggers the production of ROS. These events activate the p53/p16 pathways, leading to cellular senescence and cell-cycle arrest. UV exposure also activates AP-1, which inhibits Type I collagen synthesis and promotes the expression of MMPs. The resulting imbalance between collagen synthesis and degradation leads to the breakdown of the ECM. Additionally, pollutants like PM2.5 and PAHs penetrate the skin and induce oxidative stress and chronic inflammation via the aryl hydrocarbon receptor (AhR) pathway, further accelerating fibroblast senescence [136, 137].

### Keratinocyte senescence: barrier attrition and epidermal remodeling

Keratinocytes are the foundational constituents of the epidermal barrier, maintaining cutaneous integrity through a highly orchestrated program of proliferation, differentiation, and desquamation. The senescence of these cells precipitates a systemic collapse of barrier function, characterized by delayed turnover, epidermal thinning, and persistent xerosis. At the stratum basale, epidermal stem cells exhibit an age-related decline in proliferative potency and a shift in lineage commitment, which significantly blunts the kinetic rate of epidermal renewal and post-injury regenerative efficacy. In senescent keratinocytes, the down-regulation of terminal differentiation markers, notably filaggrin and loricrin, leads to a structurally compromised stratum corneum. This loss of barrier integrity promotes elevated transepidermal water loss and triggers the secretion of pro-inflammatory cytokines, establishing a self-perpetuating “inflamm-aging” feed-forward loop [138, 139]. Furthermore, chronic UV exposure not only induces direct genotoxic stress in basal cells but also derails their spatiotemporal differentiation programs, manifesting as either maladaptive epidermal hyperplasia or pathological atrophy [140].

### Melanocyte senescence: pigmentary dysregulation and niche exhaustion

Residing within the basal layer, melanocytes serve as essential photoprotective sentinels by synthesizing melanin to shield keratinocyte nuclei from UV-induced DNA damage. Their senescence drives the clinical hallmarks of cutaneous aging, including solar lentigines (age spots), dyspigmentation, and hair graying. Intriguingly, the process of melanogenesis is intrinsically coupled with the generation of ROS. Under chronic UV provocation, melanocytes enter a state of metabolic hyper-activation, leading to severe oxidative proteostasis in the mitochondria and endoplasmic reticulum, which ultimately necessitates the transition into senescence [141, 142]. Moreover, melanocyte homeostasis is exquisitely dependent on the paracrine support from the keratinocyte niche. Current evidence suggests that senescent keratinocytes exhibit a diminished secretome of essential growth factors; this withdrawal of microenvironmental support leads to melanocyte dysfunction or localized depletion, manifesting as hypomelanosis or erratic pigment distribution [143].

Senescence-inducing factors across different tissues exhibit both commonalities and tissue-specific characteristics. Meanwhile, various tissues interact by secreting factors such as SASP into the blood circulation, thereby mediating inter-organ crosstalk and propagating senescence systemically (Figure 2).

**Figure 2:**
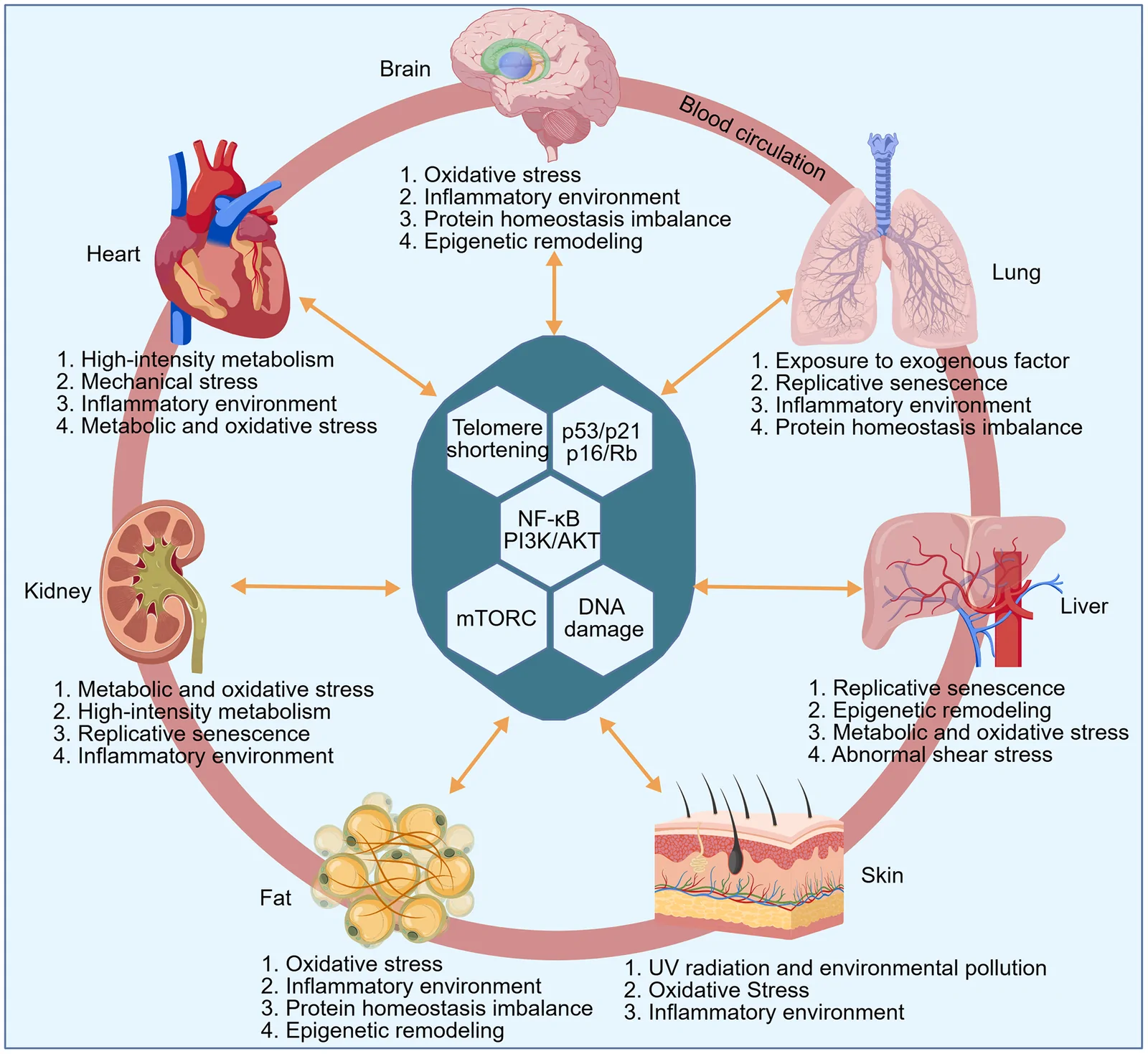
**Tissue-specific triggers of aging.** The illustration demonstrates the primary senescence-inducing factors across diverse tissues, which exhibit both commonalities and tissue-specific heterogeneity. These stressors eventually activate key aging-related pathways, such as the p16/Rb axis, leading to cellular senescence. Furthermore, various tissues interact by secreting factors like SASP into the blood circulation, thereby influencing distant organs and driving a systemic aging process.

## The double-edged sword of senolysis: balancing tissue homeostasis and functional restoration in the tissues

Current anti-aging research is primarily focused on functional rejuvenation following the clearance of senescent cells (senolysis). However, given that distinct populations of senescent cells (SnCs) maintain specific roles in preserving tissue architecture and physiological stasis—sometimes conferring pro-survival benefits (Supplementary Table 1) — therapeutic strategies must navigate a precarious equilibrium between eradicating deleterious phenotypes and preserving structural integrity.

### Diverse physiological roles of major senescent cell types in hepatic tissue

### Hepatocytes: regenerative niche vs. functional exhaustion

Targeted ablation of senescent hepatocytes significantly attenuates the secretion of SASP components, such as IL-6 and PAI-1. This mitigation relieves paracrine suppression on adjacent healthy parenchyma, thereby ameliorating insulin resistance and metabolic dysregulation [64, 144]. Furthermore, the removal of SnCs creates “biological vacancy,” providing both physical space and signaling niche support for hepatic progenitor cells to reboot regenerative programs and lowering the risk of inflammation-driven oncogenesis [145]. In the context of chronic or severe liver injury, where compensatory capacity is near its threshold, the massive clearance of senescent hepatocytes may outpace de novo cell replacement, potentially precipitating acute-on-chronic liver failure.

### Hepatic stellate cells: fibrotic resolution vs. unchecked hyperplasia

HSCs serve as the central effectors of hepatic fibrogenesis. Upon liver injury, activated HSCs proliferate and secrete ECM to form fibrotic scars, a process initially aimed at confining the injury. Nevertheless, the loss of key senescence regulators leads to sustained HSC proliferation and progressive fibrosis. Targeting the clearance of senescent HSCs at late stages has emerged as a frontline strategy for the reversal of fibrosis, as it effectively diminishes pathological ECM deposition [52].

### LSECs: microvascular rejuvenation vs. barrier rupture

LSECs form the sinusoidal wall, a critical interface for material exchange between the blood and hepatocytes, and possess robust endocytic capacity. Eliminating senescent LSECs can alleviate chronic intrahepatic inflammation and profibrotic pressure while restoring the normal expression of vascular adhesion molecules. This rejuvenation may stimulate the recruitment of healthy LSECs, recovering impaired filtration and exchange efficiency. The Risk: The clearance of LSECs may induce transient sinusoidal denudation, acutely increasing hepatic vulnerability to gut-derived toxins or micro-hemorrhage. Given the ongoing controversy surrounding the origin and replacement rate of LSECs, the “seamless substitution” of these cells remains a primary concern [13, 14, 146].

### KCs: immunomodulation vs. pathogen vulnerability

The hepatic macrophage pool primarily consists of resident KCs and monocyte-derived macrophages. Selective depletion of the pro-inflammatory senescent macrophage subpopulation directly lowers systemic hepatic “inflamm-aging” and fosters an expansion of anti-inflammatory, pro-repair macrophage subsets [13]. Technical Frontier: As the first line of immunological defense, non-selective removal of KCs precipitates a surge in susceptibility to enteric pathogens. The current challenge lies in achieving molecular granularity—precisely targeting the “senescent state” without compromising the pathogen surveillance and homeostatic endocytic functions of the healthy macrophage repertoire [95, 147].

### Functional landscapes of cell senescence in pulmonary homeostasis and disease

Senolytic interventions targeting the lung have reached the vanguard of therapeutic strategies for age-associated respiratory pathologies and fibrotic disorders. Yet, the clinical translation of these approaches is fraught with challenges, dictated by the lung's modest regenerative capacity and the intricate architecture of the blood-gas barrier. In this context, senolysis operates as a double-edged sword: while resolving pathology, its utility is circumscribed by the risk of compromising organ-scale repair.

### The AT2 progenitor niche and alveolar integrity

AT2 cells serve as the “guardians” of the alveoli. While senescent AT2 cells orchestrate fibrogenesis via the secretion of SASP factors like TGF-β and IL-11, their ablation represents a delicate balance. Targeted senolysis can rejuvenate the alveolar niche and facilitate structural remodeling [148, 149]. However, over-zealous clearance risks exhausting the progenitor reservoir, particularly in the aged or chronically injured lung, leading to defective alveolarization and metabolic derangement of pulmonary surfactants [150].

### Interstitial fibroblasts: between scaffolding and scarring

The aberrant activation and senescence of interstitial fibroblasts are the hallmarks of recalcitrant scarring. Eliminating these senescent effectors is fundamental to de-escalating ECM accumulation and restoring pulmonary compliance [70]. However, one must consider the “structural legacy” of the fibrotic matrix; in advanced disease, these cells provide critical mechanical support, and their rapid removal may provoke transient structural instability or disrupt the regenerative cues necessary for physiological healing.

### Endothelial senescence and the blood-gas barrier

VEC senescence undermines the integrity of the vast gas-exchange interface, fueling vascular resistance and chronic inflammation. While senolysis offers a potent avenue to alleviate pulmonary hypertension and dampen the inflammatory milieu [82, 151]. It carries the inherent risk of compromising the blood-gas barrier. Without concomitant endothelial repopulation, mass clearance may trigger vascular leakage, edema, and progressive rarefaction.

### The macrophage paradox: plasticity vs. senescence

Parallel to hepatic paradigms, the pulmonary macrophage pool is defined by extreme plasticity. Deciphering the “senescence-associated” detrimental state from “repair-associated” activation remains the primary hurdle for the next generation of precision senolytics [93].

### The senescence landscape in the nephron: functional diversity and therapeutic trade-offs

In the pathological landscape of renal disease, senolysis has emerged as a promising frontier for arresting CKD progression and augmenting AKI recovery. Nevertheless, the clinical utility of this approach is strictly governed by the “regenerative hierarchy” and the structural idiosyncrasies of the nephron's cellular constituents.

### RTECs: the engine of repair and rebirth

As the metabolic workhorses of the kidney, RTECs are central to post-AKI restitution. While senescent RTECs drive tubulointerstitial fibrosis via a TGF-β and CTGF-mediated SASP, their targeted removal offers a dual benefit: dampening chronic inflammation and priming the tubular niche for healthy progenitors [152, 153]. Yet, a “temporal paradox” exists aggressive senolysis during the acute injury phase may exhaust the cellular reservoir required for re-epithelialization, thereby stalling repair and precipitating acute renal collapse.

### The glomerular dilemma: podocyte fragility vs. mesangial remodeling

The glomerulus presents a unique challenge for senolytic interventions. While clearing senescent mesangial cells represents a viable strategy to halt sclerotic matrix deposition [154, 155]. podocytes represent a “non-renewable resource.” As post-mitotic cells, the loss of senescent podocytes cannot be compensated for by proliferation; thus, senolysis may inadvertently breach the glomerular filtration barrier, leading to catastrophic proteinuria and structural collapse. Furthermore, the ablation of mesangial cells must be balanced against the need to maintain the mechanical integrity of the capillary tuft, as excessive clearance may compromise glomerular hemodynamics [156].

### Fibroblasts and macrophages: conserved mechanisms of senescence

The roles of senescent renal fibroblasts and macrophages parallel those in the lung, where the pursuit of anti-fibrotic efficacy must be weighed against the potential disruption of essential scaffolding and immune-mediated healing processes [157, 158].

### Cellular senescence in the heart: functional dichotomy and therapeutic precision

Targeting senescent cells in the heart offers a potent strategy to reverse cardiac aging, yet the clinical translation of senolysis is governed by a delicate balance between removing “pathological drivers” and preserving “structural scaffolds.”

### The cardiomyocyte: contractile stability vs. numerical attrition

Senescent CMs act as paracrine foci of dysfunction, where SASP factors like MMPs and IL-6 destabilize the myocardial niche. Their removal not only curbs pathological hypertrophy but also recalibrates the electrical landscape of the heart, potentially offering a refractory shield against arrhythmias [118, 159, 160]. However, the “post-mitotic nature” of CMs renders senolysis a high-stakes gamble. The ablation of even a fraction of the CM pool risks permanent attrition of the contractile apparatus, potentially precipitating heart failure. Moreover, the heart functions as a seamless electromechanical syncytium; any focal cell loss may trigger reparative fibrosis, replacing functional myocardium with non-conductive, stiff scars [161, 162].

### The fibroblast: mastering the matrix pivot

CFs are the architects of the cardiac interstitium. While senescent CFs drive the fibrotic remodeling characteristic of heart failure with preserved ejection fraction (HFpEF), their targeted elimination can “soften” the myocardium and break the TGF-β-mediated feedback loops of senescence [163, 164]. Yet, the role of CFs is context-dependent. During acute injury or myocardial infarction, these cells provide the necessary mechanical tension to prevent ventricular wall rupture. Thus, senolytic timing is paramount: premature intervention may compromise the structural integrity of the healing heart, leading to aneurysmal complications [162].

### The endothelium: perfusion recovery vs. microvascular fragility

Senescent CECs are key contributors to the “inflammaging” of the coronary vasculature. Senolysis in the endothelial compartment holds the promise of rejuvenating angiogenesis and restoring the nitric oxide-mediated vasodilatory reserve, essential for combating ischemic heart disease [19, 119]. However, the therapeutic window is narrow. Excessive ablation of CECs may breach the endothelial barrier, resulting in myocardial edema and microvascular hemorrhage. Without robust compensatory proliferation from healthy CECs, the heart faces the risk of vascular rarefaction and a transient pro-thrombotic state, necessitating a highly controlled approach to vascular rejuvenation.

### Targeting senescence in the brain: balancing neuroprotection and circuit integrity

The brain represents a unique challenge for senolytic therapy due to the post-mitotic nature of its primary functional units and the delicate architecture of the neurovascular unit.

### The neuron: network fragility vs. proteotoxic mitigation

While the concept of the “zombie neuron” provides a target for mitigating tauopathy and SASP-induced collateral damage, the irreversible nature of neuronal loss dictates a cautious approach. Unlike peripheral tissues, the central nervous system lacks a robust regenerative reservoir. Every senolytic event in the neuronal compartment is a potential “cut” to a synaptic connectome, risking permanent cognitive erosion. The absence of high-fidelity biomarkers to distinguish between late-stage dysfunction and reversible senescence remains a major barrier to neuronal senolysis [10, 95, 127].

### The astrocyte: metabolic guardianship and blood-brain barrier homeostasis

Astrocytes are pivotal support cells of the brain, responsible for providing nutritional support, recycling neurotransmitters, maintaining the blood-brain barrier, and regulating inflammation. Eliminating senescent astrocytes that have transitioned to a reactive neuroinflammatory phenotype can significantly reduce the levels of pro-inflammatory cytokines in the brain (such as IL-1β and TNF-α), potentially facilitating the restoration of normal extracellular ionic balance and neurotrophic support. In Alzheimer’s disease models, the clearance of senescent astrocytes has been demonstrated to reduce Aβ plaque burden and tau hyperphosphorylation [131]. However, as astrocyte endfeet are essential components of the blood-brain barrier, their removal may cause localized blood-brain barrier disruption, increasing the risk of peripheral harmful substances entering the brain. Furthermore, astrocytes actively participate in the regulation of synaptic transmission; thus, non-selective clearance could impair synaptic plasticity as well as learning and memory. Given that the functions of astrocytes are highly specific across different brain regions, generalized clearance may lead to unpredictable side effects [165].

### The microglia: immunological rejuvenation and the risk of rarefaction

Senescent microglia, often characterized by a loss of motility and impaired phagocytic efficiency, are prime candidates for therapeutic targeting. Clearing these cells may reset the neuroinflammatory milieu and halt aberrant synaptic pruning [133, 135]. Yet, this “reset” comes with a cost: a temporary depletion of the brain's innate immune shield, potentially lowering the threshold for opportunistic infections. The challenge lies in the phenotypic plasticity of microglia; identifying the transition from “protective activation” to “pathological senescence” is essential to avoid interfering with necessary immune responses [166].

Current paradigms suggest that the most viable neuroprotective strategy is not the broad elimination of cells, but a tiered approach: high-precision senolysis of inflammatory microglia coupled with senomorphic modulation of the SASP. By neutralizing the “bystander effect” of senescence without sacrificing neuronal numbers, we can potentially preserve the structural and functional continuity of the aging brain.

### Functional impact and therapeutic implications of major senescent cell types in adipose tissue

### Adipocyte senescence and the risk of systemic lipid spillover

Adipocytes represent terminally differentiated cells specialized for triglyceride storage and adipokine secretion. The targeted clearance of senescent or dysfunctional adipocytes, characterized by insulin resistance and aberrant lipolysis, can significantly optimize the local microenvironment. This intervention reduces the secretion of pro-inflammatory SASP components, such as IL-6, MCP-1, and PAI-1, thereby alleviating chronic adipose tissue inflammation. Furthermore, such clearance may create physical space and metabolic niches for healthy adipocytes, potentially enhancing the functionality of the remaining cell population through optimized lipid turnover [45]. However, several risks must be considered. The abrupt rupture of a substantial number of adipocytes may trigger a massive release of free fatty acids into the systemic circulation, exacerbating lipid spillover and subsequent insulin resistance in peripheral organs such as the liver and skeletal muscle. Additionally, large-scale non-selective removal may temporarily disrupt systemic homeostasis of critical hormones, including leptin and adiponectin.

### Senescent macrophages as primary drivers of chronic metabolic inflammation

The elimination of senescent ATMs is among the most direct and potent strategies for mitigating chronic low-grade inflammation, leading to rapid improvements in both local and systemic insulin sensitivity. Clearing these cells optimizes the immune microenvironment by favoring macrophage polarization toward the anti-inflammatory M2 phenotype and promoting tissue repair. Given that the SASP of senescent ATMs recruits additional monocytes and induces secondary paracrine senescence, their clearance effectively interrupts this self-perpetuating detrimental cycle [167, 168].

### Adipocyte progenitor senescence and the exhaustion of regenerative capacity

APCs and ASCs, localized within the stromal vascular fraction, are responsible for de novo adipogenesis and constitute the foundation of adipose tissue plasticity and health. The clearance of senescent APCs—which have lost their adipogenic differentiation potential—may provide the remaining healthy APCs with the requisite space and inductive signaling to proliferate and differentiate into functional, insulin-sensitive adipocytes. This process is critical for achieving healthy adipose tissue hyperplasia rather than pathological adipocyte hypertrophy. Moreover, since senescent APCs are prone to secreting pro-fibrotic factors, their removal can reduce adipose tissue fibrosis [123, 124]. Nevertheless, as APCs represent the sole source of regeneration, excessive or frequent clearance may irreversibly exhaust the progenitor pool. This could lead to a total loss of the tissue capacity for healthy expansion in response to caloric surplus, ultimately accelerating metabolic deterioration.

### Functional consequences of senescent cell accumulation in the skin and therapeutic modulation

### Senescent dermal fibroblasts as primary drivers of structural degradation

Dermal fibroblasts constitute the functional core of cutaneous structural integrity, responsible for the synthesis of ECM components, including collagen and elastin. The accumulation of senescent fibroblasts represents the fundamental structural etiology of skin wrinkling and loss of tensile strength. Targeted elimination of these cells significantly mitigates the secretion of the SASP, particularly IL-6 and MMPs, which are the primary agents of collagen degradation and the inhibitors of de novo collagen synthesis [15]. This mechanism stands as the most potent intervention for restoring skin thickness, enhancing elasticity, and reducing rhytids. Furthermore, the clearance of senescent fibroblasts re-establishes a favorable niche for healthy fibroblast populations, facilitating the secretion of functional ECM. Clinical evidence supports this approach; topical application of senolytic agents, such as fisetin, has demonstrated efficacy in improving human skin health parameters in clinical trials [20, 136].

### Senescence of cutaneous immune cells and the erosion of immunological surveillance

Langerhans cells serve as the primary immunological sentinels within the skin, and their senescence leads to a progressive decline in antigen-presentation efficacy. Theoretically, the targeted removal of senescent Langerhans cells may stimulate the replenishment of functional, immunocompetent immune cells, thereby rejuvenating cutaneous immunosurveillance and tolerance. However, the acute clearance of these sentinels involves significant physiological trade-offs. The temporary depletion of the immune cell pool may transiently impair the localized defense against pathogens, such as viruses and fungi, thereby increasing the susceptibility to opportunistic infections during the remodeling phase.

## Prevention and intervention of senescence

### Exercise-mediated anti-aging

Exercise can delay or inhibit cellular senescence across various tissues [25, 169, 170]. Aging is a complex biological process involving multiple organs and systems; although irreversible, its progression can be effectively delayed through scientific interventions. In recent years, with the deepening understanding of the molecular mechanisms of aging, researchers have gradually revealed the systemic mechanisms by which various lifestyle interventions delay senescence. As a core intervention, the molecular mechanism of exercise in delaying aging has recently been systematically elucidated. A study published in cell first clarified that long-term regular exercise not only reshapes metabolic and immune homeostasis and delays immune cell senescence, but also induces the production of the endogenous molecule “betaine.” By recruiting healthy volunteers for a 25-day study comparing long-term regular exercise with acute exercise, combined with multi-omics analysis, the study found that chronic exercise significantly upregulates renal betaine synthesis. This synthesis depends on the two-step oxidative metabolism of mitochondrial choline, with choline dehydrogenase (CHDH) serving as the key rate-limiting enzyme that is upregulated by exercise induction [25]. As an endogenous metabolite, betaine effectively inhibits the release of pro-inflammatory factors by specifically binding to and inhibiting the activity of TANK-binding kinase 1 (TBK1), a hub kinase of innate immunity, thereby blocking the activation of downstream IRF3/NF-κB signaling pathways. In aged mouse models, oral administration of betaine significantly improved multiple organ function indicators, including metabolic capacity, renal function, motor coordination, and cognitive function, with the most significant anti-aging effects observed in the kidneys and skeletal muscle. This discovery not only provides a molecular footnote to the ancient perception of “exercise as the fountain of youth” but also initiates a new R&D paradigm based on “endogenous metabolites mediating exercise benefits,” offering a potential anti-aging alternative for elderly populations unable to tolerate long-term high-intensity exercise.

### Management of blood glucose and blood pressure

Regulating glycemic levels, blood pressure, and lipid profiles is fundamental to maintaining “core metabolic homeostasis.” As previously discussed in the context of cellular senescence, chronic hyperglycemia facilitates the formation of AGEs via glycation reactions. These “senescent macromolecules” accumulate under hyperglycemic conditions and, by binding to their receptor, activate NADPH oxidase-derived oxidative stress. This process promotes the generation of ROS, subsequently upregulating the expression of Cdk5 and CD36 genes, which induces macrophage foam cell formation—the primordial step in atherosclerosis. Recent studies have demonstrated that glucose-dependent insulinotropic polypeptide can suppress AGE-induced oxidative stress and foam cell formation by activating the AMPK signaling pathway, highlighting the critical role of glycemic regulation in vasoprotection. Concurrently, hypertension and dyslipidemia drive vascular aging through the common pathway of endothelial dysfunction. Excessive ROS reduce the bioavailability of NO and impair endothelial nitric oxide synthase function, leading to vascular inflammation and compromised vasodilation. Furthermore, ox-LDL triggers endothelial injury and foam cell formation, thereby accelerating the atherosclerotic process. Hypertension exacerbates vascular dysfunction by disrupting the equilibrium between vasodilators and vasoconstrictors while intensifying oxidative stress [171]. Clinical evidence suggests that 12-week lifestyle interventions, such as dance or balance training, significantly improve physical function in the elderly and lead to substantial reductions in systolic blood pressure and insulin resistance. These findings underscore the integrated regulatory role of lifestyle modifications on key metabolic parameters [172]. Pharmacological control of blood glucose and blood pressure represents a viable anti-aging strategy. Common agents, including the antidiabetics Metformin and Empagliflozin and antihypertensives such as Angiotensin II receptor blockers, exert anti-aging effects not only through their primary metabolic and hemodynamic benefits but also by directly modulating pro-aging molecular cascades. Specifically, Metformin acts by suppressing the SASP to alleviate senescent phenotypes; Empagliflozin targets the PI3K/Akt/p21 axis to mitigate hepatic aging [173, 174]; and Angiotensin II antagonists counteract the mitochondrial impairment and subsequent cellular senescence typically driven by Angiotensin II [175].

### Modulation of gut microbiota and microbial metabolism

Dysbiosis of the gut microbiota is inextricably linked to age-related metabolic disorders, including obesity, NAFLD, and dyslipidemia [176, 177]. During aging, the gut microbiota often transitions into a state of dysbiosis characterized by a reduced abundance of short-chain fatty acid (SCFA)-producing taxa. Intermittent fasting and probiotic interventions can independently or synergistically promote the enrichment of core SCFA-producing strains, such as Anaerostipes hadrus, Roseburia intestinalis, and Akkermansia muciniphila. This enrichment restores gut microbial balance and metabolic health in aging hosts. Furthermore, the resulting systemic improvement in aging status reciprocally stabilizes the beneficial microbial ecosystem, establishing a virtuous cycle rather than a unidirectional causal link. Furthermore, a 30-day clinical trial of 16:8 time-restricted eating (TRE) in healthy volunteers revealed that TRE significantly reduced the frequency of circulating senescent T cells (CD4+CD27-CD28-), with more pronounced effects in individuals over 30 years old. This dietary regimen also enhanced T-cell receptor diversity, elevated levels of anti-inflammatory and anti-aging serum metabolites, and upregulated the abundance of longevity-associated microbiota [178].

Integrating exercise with TRE has demonstrated synergistic benefits in improving body mass index and blood pressure among postmenopausal women, surpassing the efficacy of exercise alone. This suggests that multidimensional lifestyle interventions represent a pivotal direction for future anti-aging strategies. Within the theoretical framework of the hallmarks of aging, physical exercise, metabolic control, and emerging lifestyle interventions achieve anti-aging effects at molecular, cellular, and systemic levels by targeting core hallmarks, including genomic instability, epigenetic alterations, mitochondrial dysfunction, cellular senescence, and chronic inflammation [176].

In summary, the paradigm of preventing aging has evolved from the exploration of single modalities into a systematic endeavor involving multidimensional combined interventions. By stimulating endogenous repair mechanisms (e.g., the exercise-induced betaine-TBK1 axis), maintaining metabolic homeostasis (through the regulation of glucose, blood pressure, and lipids), and expanding emerging therapeutic targets (e.g., the gut-muscle axis and TRE-induced immune rejuvenation), these strategies provide an increasingly robust theoretical foundation and practical roadmap for achieving healthy aging.

## Therapeutic modalities for targeting senescent cells

### From broad-spectrum cytotoxicity to refined specificity

The field of small-molecule senolytics is undergoing a paradigm shift, transitioning from first-generation agents characterized by broad-spectrum but non-specific toxicity toward next-generation compounds designed for enhanced selectivity and safety. In 2015, Kirkland and colleagues at the Mayo Clinic pioneered a landmark senolytic strategy utilizing a combination of dasatinib and quercetin (D+Q). This dual regimen selectively induces apoptosis in senescent cells and has been demonstrated to effectively reduce senescent cell burden while extending lifespan in murine models. Mechanistically, dasatinib functions by inhibiting Src kinases and BCL-2 family signaling, while quercetin disrupts PI3K/AKT pathways to dismantle the pro-survival networks that shield senescent cells from apoptosis (Figure 3) [17, 179]. Systemic administration of D+Q has been shown to improve cardiac function, alleviate vascular stiffness, and enhance physical endurance in aged mice. Notably, D+Q therapy was the first senolytic regimen to enter clinical evaluation (Table 1), with early-phase trials conducted in patients with idiopathic pulmonary fibrosis [18]. However, significant challenges persist, including the lack of a singular molecular target, potential off-target effects, suboptimal oral bioavailability, and inconsistent efficacy observed in certain clinical contexts [18, 180].

**Figure 3:**
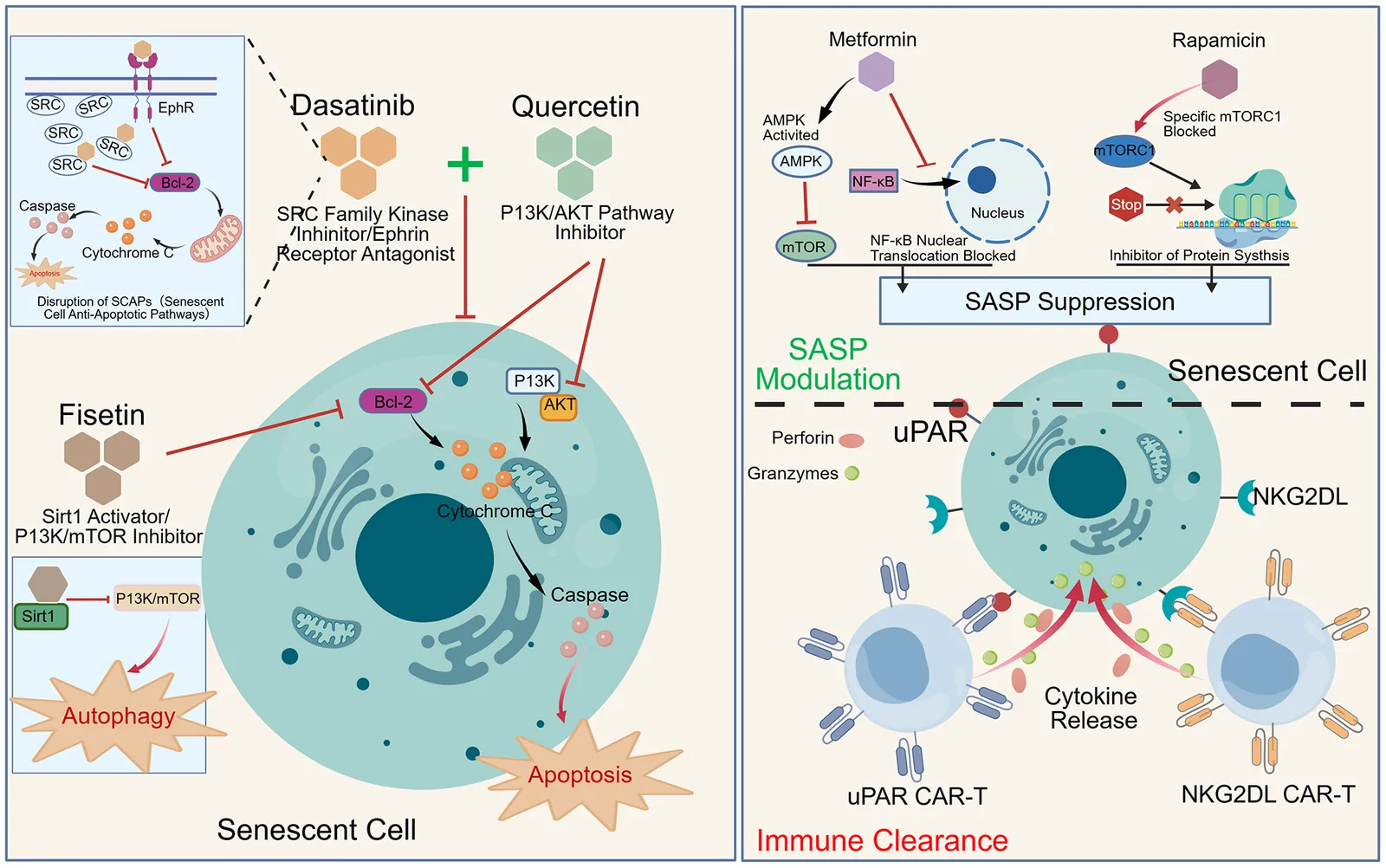
**Mechanistic landscape of pro-longevity strategies.** This figure illustrates the mechanistic action of key small-molecule drugs in clearing senescent cells, alongside immunotherapy strategies designed for the same purpose. Furthermore, it depicts the mechanisms by which anti-aging agents, such as SASP inhibitors, reduce the secretion of the senescence-associated secretory phenotype to achieve therapeutic anti-aging effects.

**Table 1:** Summary of current clinical trials on anti-aging interventions.

**Intervention**	**Target condition**	**Clinical trials.gov (NCT ID)**	**Phase**	**Key findings / status**	**Potential side effects**
**Dasatinib + Quercetin**	Idiopathic pulmonary fibrosis	NCT02874989	Phase I	First human trial of senolytics. No lung function alleviation but improved physical mobility.	Mild gastrointestinal discomfort, skin rash, and intermittent headache.
**Dasatinib + Quercetin**	Diabetic Kidney Disease	NCT02848131	Phase II	Significant reduction of p16 and p21 in adipose and skin tissues; decreased plasma SASP levels.	Renal monitoring (well-tolerated); risk of anemia.
**Dasatinib + Quercetin**	Alzheimer's disease	NCT04685590 (SToMP-AD)	Phase II	Assessment of CSF penetrance and safety; demonstrated favorable preliminary tolerability.	Central nervous system monitoring; potential cytopenia.
**Fisetin**	Frailty and inflammation	NCT03430037	Phase II	AFFIRM trial: efficacy of interventions on inflammation and frailty in older adults.	Generally safe; potential for mild dyspepsia at high dosages.
**Fisetin**	Chronic kidney disease	NCT03325322	Phase II	Effects of Fisetin on stromal cell, renal function, inflammation, and physical performance.	Generally safe; potential for mild dyspepsia at high dosages.
**Metformin**	Alzheimer's disease	NCT01965756	Phase II	Short-term use alters systemic and CSF inflammatory proteomes with preliminary cognitive improvements	Nausea and diarrhea.
**Metformin**	Age-related multimorbidity	TAME Study	Phase III (planning)	First global trial with "aging" as primary endpoint to delay multiple age-related chronic diseases.	Nausea/diarrhea; rare lactic acidosis; risk of vitamin B12 deficiency.
**Rapamycin**	Ovarian Aging	NCT05836025	Phase II	Efficacy of intervention in delaying ovarian aging and preserving ovarian reserve.	Oral ulcers, hyperlipidemia, peripheral edema, and potential delayed wound healing.

Next-generation senotherapeutic agents have demonstrated superior efficacy and improved safety profiles. Fisetin, a naturally occurring flavonoid, has emerged as a potent senolytic capable of reducing senescent cell load and extending healthspan in aged mice [20]. Its primary modes of action involve the modulation of antioxidant pathways and the inhibition of anti-apoptotic proteins (Figure 3); furthermore, as a natural product, it generally exhibits favorable oral safety and tolerability, though its precise molecular targets warrant further elucidation (Table 1). Additionally, cardiac glycosides such as ouabain and digoxin derivatives have shown high potency in eliminating senescent human adipocytes and fibroblasts. By inhibiting the membrane Na+/K+ ATPase pump, these agents disrupt intracellular ionic homeostasis, thereby triggering apoptosis in senescent populations. While particularly effective against recalcitrant cell types like senescent adipocytes, their narrow therapeutic index and risk of cardiotoxicity necessitate extreme clinical caution [181]. Beyond small molecules, the FOXO4-DRI peptide has been shown to rapidly clear senescent cells in mice, significantly reversing functional decline in renal capacity and fur density induced by both chemotherapy and chronological aging [148, 182].

An alternative therapeutic strategy involves the use of senomorphics—agents that do not induce cell death but instead suppress the harmful SASP (Figure 3). Modulators such as rapamycin, metformin, and JAK/STAT inhibitors have effectively mitigated senescence-related chronic inflammation and tissue damage in various animal models. Clinical data for rapamycin and metformin already suggest potential geroprotective effects (Table 1). Recent mechanistic insights reveal that the accumulation of cytoplasmic chromatin fragments in senescent cells is a critical driver of the cGAS-STING pathway, which fuels chronic inflammation and SASP production, thereby providing a novel target for senomorphic intervention [80]. For instance, local application of JAK inhibitors has been shown to neutralize SASP, suppress cartilage degradation, and promote regenerative processes in osteoarthritis models, validating the feasibility of “neutralizing toxicity without cell elimination” [183]. Modulating senescent cells without inducing cell death is of paramount importance for the protection of specific tissues and organs. In certain cell populations with negligible regenerative capacity—such as AT2 cells, podocytes, cardiomyocytes, and neurons—their clearance could lead to irreversible functional loss, an outcome that is unacceptable in anti-aging research. Therefore, employing senomorphic strategies to modulate the secretome of these cells, rather than eliminating them, represents a more prudent and stable therapeutic approach for these vital tissues.

### Immunotherapeutic approaches for targeted senolysis

The advancement of CAR-T cell technology has recently catalyzed a breakthrough in precision senolysis. In 2020, this modality was first adapted for anti-aging intervention through the engineering of CAR-T cells targeting the uPAR. This strategy successfully eliminated senescent cells in the liver and lungs of murine models induced by high-fat diets, chemotherapy, or natural aging. Beyond mitigating NASH and pulmonary fibrosis, the intrinsic immune memory of T cells enabled a one-time intervention to yield persistent therapeutic benefits [21]. Building on this foundation, research from Cold Spring Harbor Laboratory in 2024 further validated that uPAR-targeted strategies could selectively ablate senescent populations, thereby restoring metabolic vitality in aged mice and decelerating the aging process in younger cohorts [22]. Most recently, in November 2025, studies identified uPAR-positive cells as pivotal drivers of intestinal senescence; their precision clearance was shown to preserve intestinal stem cell function and effectively prevent or reverse age-related intestinal dysfunction [184]. Complementary to cell-based therapies, novel bridging strategies, such as the E16-uPA24 small-molecule peptide, are being developed to enhance the recognition efficiency of endogenous immune cells toward senescent targets, thereby alleviating pathological hallmarks [185]. Diversification of molecular targets is critical for broader clinical application. In August 2023, researchers leveraged the aberrant surface expression of NKG2D ligands (NKG2DL) on senescent cells to develop NKG2D-based CAR-T cells (Figure 3). This approach demonstrated robust broad-spectrum senolytic capacity and an optimized safety profile in non-human primates, suggesting a versatile therapeutic potential that transcends tissue-specific markers [23]. Furthermore, in the context of fibrotic pathologies, CAR-T cells targeting platelet-derived growth factor receptor beta (PDGFR-β) have shown remarkable efficacy in models of chronic kidney disease. Compared to traditional fibroblast activation protein targeting, the PDGFR-β strategy facilitates a more potent synergistic intervention across renal, myocardial, and vascular fibrosis. Preliminary safety assessments indicate a lack of significant “on-target, off-tumor” toxicity, providing a robust pharmacological foundation for subsequent clinical translation. [152]. However, the clinical translation of CAR-T cell therapy is significantly hampered by its prohibitive manufacturing costs, the risk of potential cytokine release syndrome (CRS), and potential off-target toxicities, which may lead to collateral organ damage. In recent years, bispecific antibodies have emerged as pivotal tools in cancer immunotherapy [186]. Transitioning from a CAR-T-based approach to a dose-titratable bispecific antibody strategy could substantially mitigate these risks. Such a shift offers a more controllable and cost-effective framework, thereby facilitating the clinical translation of senotherapeutic interventions.

Despite the translational progress of anti-aging drugs in clinical settings, nearly all candidates are associated with certain side effects. While clinical outcomes for some ongoing anti-aging studies remain undisclosed, potential adverse effects can be extrapolated based on their safety profiles in the treatment of other diseases (Table 1).

## Concluding and Perspectives

Substantial progress has been made in characterizing the triggers and heterogeneous functions of senescent cells across major physiological systems. Beyond the organs previously discussed, the accumulation of senescent cells and their evolving functional landscapes have been extensively documented in the ovaries [187], pancreas [12], small intestine [188], thymus and immune compartments [189], prostate [190], bone tissue [191], and skeletal muscle [192].

Elucidating the tissue-specific mechanisms that trigger cellular senescence is paramount for developing robust strategies to delay biological aging and prevent premature age-related pathologies. It is increasingly clear that cellular senescence is not a terminal state driven by a single pathway, but rather a convergent outcome of diverse cell-intrinsic and extrinsic stressors. First, the DNA damage response remains a canonical driver of senescence. Chronic genotoxic stress—emanating from ultraviolet radiation, chemical mutagens, and endogenously produced ROS—compromises genomic stability. Bolstering antioxidant defenses or enhancing DNA repair fidelity could potentially mitigate the accumulation of molecular lesions, thereby pre-empting the onset of premature senescence at its source. Second, telomere attrition serves as an intrinsic “mitotic clock” that governs replicative potential. Mounting evidence suggests that lifestyle modifications (such as optimized nutrition and regular exercise) or pharmacological preservation of telomerase activity may alleviate replicative senescence triggered by critical telomere shortening. Furthermore, the loss of proteostasis, mitochondrial dysfunction, and dysregulated nutrient-sensing signaling, specifically involving the mTOR and AMPK pathways, alongside aberrant stress-sensing mechanisms, represent critical trajectories that accelerate the systemic progression of cellular senescence.

As the field of geroscience evolves, it has become evident that the biological significance of senescent cells depends less on their presence than on their context-specific “functional roles.” Current molecular markers, while useful for identification, fail to discriminate between functionally distinct senescent subpopulations. Consequently, the focus of research is shifting from “identifying the senescent state” to “evaluating functional pathogenicity,” prioritizing the targeting of cell clusters that actively disrupt tissue homeostasis or drive specific disease ontologies. This shift necessitates an evolution in senolytic strategies toward “non-toxic precision clearance,” requiring intervention tools to act as molecular scalpels capable of distinguishing deleterious cells from neutral or even beneficial ones. For instance, senescent β-cells in the pancreas exhibit enhanced insulin secretion compared to their non-senescent counterparts [12], suggesting that their indiscriminate clearance might paradoxically impair glucose metabolism. Similarly, while acute depletion of senescent endothelial cells, which maintain the blood–organ barrier, could risk irreversible vascular damage, evidence of benefit from their clearance in other contexts [193]. We have previously discussed the biphasic functional transition of senescent LSECs: their functional activity initially undergoes a compensatory increase during the early stages of aging, followed by a progressive decline as senescence advances. This phenomenon suggests that senolytic interventions cannot rely on a simplified “one-size-fits-all” approach based solely on chronological age. Instead, it underscores the critical need for a more sophisticated strategic framework—one that identifies the optimal timing and methodologies to target endothelial senescence based on real-time functional status rather than mere temporal progression. The biological aging process is fundamentally non-linear. Recent studies of the immune system reveal that peripheral T cell senescence does not progress steadily with chronological age, highlighting the importance of stage-specific immune modulation [26]. Furthermore, proteomic aging across different tissues follows distinct, non-linear trajectories; notably, age 30 has been identified as a critical susceptibility window where proteomic shifts in body fluids begin to drive vascular and systemic aging [27]. Organismal aging exhibits pronounced tissue heterogeneity and asynchronous timelines. Rather than an isolated local phenomenon, aging is a systemic process characterized by the coordinated evolution of multiple organs [194]. In particular, SASP factors, such as GAS6, TGF-β, and IL-23R, act as mediators of inter-organ communication, triggering “bystander effects” in distal tissues via the systemic circulation [27, 195, 196]. Research on systemic aging has identified the aorta as one of the earliest organs to manifest senescent features. Specifically, Growth Arrest Specific 6 (GAS6), a vasculo-angiogenic factor secreted by the aorta, is upregulated with age and enters the bloodstream to accelerate senescence in the liver, kidneys, and other tissues. Similarly, liver-derived TGF-β propagates through the circulation to induce renal senescence, an effect that can be mitigated by TGF-β receptor inhibitors. Consequently, dynamic monitoring of circulating SASP levels is pivotal for deciphering the systemic trajectory of aging and provides a theoretical framework for developing cross-organ anti-aging interventions.

The future of this field is pivoting toward a profound integration of precision clearance and prospective intervention. As research continues to unveil the heterogeneous aging trajectories of different organs and tissues, single-cell sequencing has become instrumental in identifying stage-specific anti-aging targets, providing a scientific benchmark for the optimal timing of senolysis. This “optimality” necessitates the dynamic monitoring of tissue microenvironments to implement interventions within specific temporal windows. By leveraging strategies such as uPAR-targeted CAR-T cell therapy and lipid nanoparticle delivery systems for *in situ* CAR-T generation, the field is evolving from “broad-spectrum clearance” toward “precision recognition.” Utilizing specific targets and transient mechanisms allows for the selective elimination of deleterious senescent cells while rigorously safeguarding non-renewable cells, such as cardiomyocytes and neurons, as well as beneficial senescent cells essential for physiological repair. This paradigm imposes more rigorous requirements for target discovery. By leveraging single-cell sequencing to identify high-value targets and integrating real-time dynamic monitoring of the tissue microenvironment, we can strategically refine our senolytic approach—specifically by transitioning to bispecific antibodies. This integrated framework ensures the elimination of deleterious senescent cells with surgical precision at the optimal therapeutic window. A more transformative perspective involves “sequential intervention” strategies. This approach recognizes that senescence is not a synchronized systemic event but is often initiated in specific “pioneer organs” before spreading via systemic “contamination.” By constructing detailed “organ-aging atlases,” we can identify the fuse nodes, tissues under high metabolic or mechanical stress that ignite the initial senescence signals. Transitioning from treating end-stage systemic decline to intercepting these primary signals allows for a paradigm shift from “treating established disease” to “intercepting at the source.”

In summary, elucidating the molecular triggers of cellular senescence is not only fundamental to understanding the core biological laws of life but also provides the precision targets required for homeostatic maintenance. The paradigm of senescence intervention is undergoing a fundamental transformation from a crude “anti-state” approach to a sophisticated “systemic management” framework. By integrating dynamic functional diagnostics, spatiotemporal precision therapies, and early sequential prevention, we are entering a more scientific, rational, and hopeful era in the quest to extend human healthspan.

## Supplementary Material

Supplementary Table 1
