# Supplementary material for: Cellular senescence: from pathogenic mechanisms to precision anti-aging interventions
Source: Aging (Albany NY). 2026 May 4;18(1):421–54. doi: 10.18632/aging.206375 (PMC13632854; doi:10.18632/aging.206375)
Supplement: Supplementary file 1 [file aging-18-1-206375-s001.docx]

Supplementary Table 1. Benefits and harms of tissue-specific senescent cell elimination.

| **Tissue** | **Cell type** | **Effects of clearing senescent cells** | | **References** |
| --- | --- | --- | --- | --- |
|  |  | Beneficial | Harmful |  |
| Liver | Hepatocytes | Attenuating SASP;  Ameliorating insulin resistance | Precipitating acute liver failure | 65, 144, 145 |
|  | HSCs | Attenuating liver fibrosis |  | 53 |
|  | LSECs | Attenuating liver fibrosis;  Alleviating chronic inflammation;  Restore impaired endocytic function | Damage to the hepatic sinusoidal barrier | 13, 14, 146 |
|  | KCs | Attenuating SASP;  Attenuating liver fibrosis | Compromise immune defenses | 13, 48, 147 |
| Lung | AT2 | Attenuating SASP;  Reversing fibrosis;  Optimize pulmonary function | Impair alveolar structural repair | 148, 149, 150 |
|  | Fibroblasts | Reduce the accumulation of extracellular matrix (ECM) components;  Reversing fibrosis;  Restore the elasticity and ventilatory function of lung tissues | Lead to transient instability of the lung microarchitecture | 71 |
|  | VECs | Restore pulmonary vasodilation function;  Reduce vascular resistance | Induce pulmonary edema;  Aggravate blood vessel pruning | 83; 151 |
| Kidney | RTECs | Alleviating chronic inflammation;  Attenuating fibrosis; | Impaired tubular regeneration | 152, 153 |
|  | Glomerulus | Attenuate glomerular sclerotic changes;  Reduce the deposition of abnormal extracellular matrix | Trigger the albuminuria;  Impair the glomerular filtration barrier. | 154, 155, 156 |

| **Tissue** | **Cell type** | **Effects of clearing senescent cells** | | **References** |
| --- | --- | --- | --- | --- |
|  |  | Beneficial | Harmful |  |
| Heart | Cardiomyocyte | Reduce the risk of arrhythmia;  Alleviate pathological myocardial hypertrophy | Cause depletion of sarcomeres/myofibrils;  Chronic heart failure | 118, 159, 160, 161, 162 |
|  | Fibroblast | Decrease cardiac muscle rigidity;  Enhance left ventricular diastolic filling | Cause progressive thinning of the ventricular myocardium;  Development of an aneurysm. | 162, 163,164 |
|  | CECs | Stimulate neovascularization;  Restore endothelium-dependent vasodilation;  Reduce leukocyte infiltration and vascular inflammation. | Cause microvascular pruning/loss; | 19,119 |
| Brain | Neuron | Optimize the neuronal niche for neighboring cells. | Cause long-term functional sequelae across cognitive and motor domains. | 10, 48. 127 |
|  | Astrocytes | Alleviating chronic inflammation;  Reduce Aβ plaque burden and tau hyperphosphorylation. | Induce localized BBB compromise and increased paracellular permeability. | 131, 165 |
|  | Microglia | Restore the homeostatic clearance of proteinaceous aggregates. (Aβ and hyperphosphorylated tau);  Preserve synaptic integrity and maintain excitatory-inhibitory balance. | Impair the brain's innate immune surveillance and defense mechanisms. | 133,135, 166 |

| **Tissue** | **Cell type** | **Effects of clearing senescent cells** | | **References** |
| --- | --- | --- | --- | --- |
|  |  | Beneficial | Harmful |  |
| Fat | Adipocyte | Attenuating SASP; |  | 45 |
|  | Macrophage | Attenuation of meta-inflammation;  Amelioration of insulin resistance |  | 167, 168 |
|  | APCs/ASCs | Attenuation of adipose tissue fibrosis | Compromising lipid storage capacity;  Impaired adipogenic potential | 123,124 |
| Skin | Dermal fibroblasts | Attenuating SASP;  Promoting a homeostatic matrix composition |  | 15 |
|  | Langerhans cells | Promoting immune tolerance/homeostasis | Compromised antimicrobial defense | 20, 136 |

Note: HSCs: Hepatic Stellate Cells, LSECs: Liver Sinusoidal Endothelial Cells, KCs: Kupffer Cells, AT2: Type II Alveolar Cell, VECs: Vascular Endothelial Cells, RTECs: Renal Tubular Epithelial Cells, CECs: Cardiac Endothelial Cells, APCs/ASCs: Adipocyte Progenitor Cells /Adipose-Derived Stem Cells.
